# *Staphylococcus aureus* Sortase A-Mediated Incorporation of Peptides: Effect of Peptide Modification on Incorporation

**DOI:** 10.1371/journal.pone.0147401

**Published:** 2016-01-22

**Authors:** Silvie Hansenová Maňásková, Kamran Nazmi, Wim van ‘t Hof, Alex van Belkum, Nathaniel I. Martin, Floris J. Bikker, Willem J. B. van Wamel, Enno C. I. Veerman

**Affiliations:** 1 Department of Periodontology and Oral Biochemistry, Academic Centre for Dentistry Amsterdam (ACTA), University of Amsterdam and VU University Amsterdam, Amsterdam, The Netherlands; 2 Department of Medical Microbiology and Infectious Diseases, Erasmus MC, Rotterdam, The Netherlands; 3 Department of Medicinal Chemistry and Chemical Biology, Utrecht Institute for Pharmaceutical Sciences, Utrecht University, Utrecht, The Netherlands; Rockefeller University, UNITED STATES

## Abstract

The endogenous *Staphylococcus aureus* sortase A (SrtA) transpeptidase covalently anchors cell wall-anchored (CWA) proteins equipped with a specific recognition motif (LPXTG) into the peptidoglycan layer of the staphylococcal cell wall. Previous *in situ* experiments have shown that SrtA is also able to incorporate exogenous, fluorescently labelled, synthetic substrates equipped with the LPXTG motif (K(FITC)LPETG-amide) into the bacterial cell wall, albeit at high concentrations of 500 μM to 1 mM. In the present study, we have evaluated the effect of substrate modification on the incorporation efficiency. This revealed that (i) by elongation of LPETG-amide with a sequence of positively charged amino acids, derived from the C-terminal domain of physiological SrtA substrates, the incorporation efficiency was increased by 20-fold at 10 μM, 100 μM and 250 μM; (ii) Substituting aspartic acid (E) for methionine increased the incorporation of the resulting K(FITC)LPMTG-amide approximately three times at all concentrations tested; (iii) conjugation of the lipid II binding antibiotic vancomycin to K(FITC)LPMTG-amide resulted in the same incorporation levels as K(FITC)LPETG-amide, but much more efficient at an impressive 500-fold lower substrate concentration. These newly developed synthetic substrates can potentially find broad applications in for example the *in situ* imaging of bacteria; the incorporation of antibody recruiting moieties; the targeted delivery and covalent incorporation of antimicrobial compounds into the bacterial cell wall.

## Introduction

*Staphylococcus aureus* infections are complex events that occur at the interface of the host and the pathogen [1]. From a bacterial perspective, infections require expression of virulence factors including cell wall-anchored (CWA) proteins [2]. The C-terminal region of staphylococcal CWA proteins contains a cluster of positively charged amino acids, which positions the CWA protein precursors to the cytoplasmic side of the bacterial cell membrane, and a hydrophobic transmembrane domain that immobilizes CWA protein precursors within the cell membrane, facilitating further processing by membrane-associated sortase A (SrtA) [3, 4]. The SrtA enzyme recognizes the LPXTG motif of these proteins and catalyses their cleavage between threonine and glycine residues [5]. Next, SrtA catalyses a transpeptidation reaction by which the proteins are covalently linked to the peptidoglycan precursor lipid II [6–8]. Then, the peptidoglycan precursor moiety that is coupled to lipid II is incorporated following transglycosylation. SrtA is therefore closely involved in the control of *S*. *aureus* virulence [9–12].

In previous studies, it has been demonstrated that endogenous SrtA is also able to incorporate exogenously added K(FITC)LPETG-amide, a fluorescently labelled artificial SrtA substrate, into the cell wall of *S*. *aureus* strains and of other Gram-positive bacteria. This required, however, high substrate concentrations, in the mM range [13, 14]. The present study was aimed at the development of novel SrtA substrates exhibiting improved incorporation efficiency. In our view, such substrates might find applications in for example *in situ* imaging, the incorporation of antibody recruiting moieties, and the targeted delivery and covalent incorporation of antimicrobial compounds into the bacterial cell wall.

In the present study a number of novel SrtA substrates were developed and their covalent incorporation into the staphylococcal cell wall was characterised *in situ*. Three different types of substrate modifications were explored: (i) variants of the K(FITC)LPETG-amide, which had been elongated with sequences derived from physiological SrtA substrates—sequences containing a hydrophobic membrane spanning domain linked to a stretch of positively charged amino acids; (ii) a substrate (K(FITC)LPMTG-amide) in which glutamate (E) was substituted for methionine (M), which appeared to be better processed by SrtA *in vitro* [15] and (iii) K(FITC)LPMTG-amide conjugated to vancomycin, designed to potentially improve the targeting to lipid II, the primary acceptor for physiological SrtA substrates in *S*. *aureus*.

## Materials and Methods

### Solid-phase peptide synthesis

SrtA substrates were manufactured by solid-phase peptide synthesis using 9-fluorenyl-methoxycarbonyl (Fmoc)-chemistry with a Syro II synthesizer (Biotage EU Customer Service, Uppsala, Sweden) essentially as described previously [13, 14, 16]. Since the presence of a carboxylic acid group at the C-terminus of the SrtA recognition motif (LPXTG) may inhibit SrtA activity, an amide group was incorporated at this position [17]. Peptide synthesis grade solvents were obtained from Actu-All Chemicals (Oss, The Netherlands), the preloaded NovaSyn TGA resins from NovaBiochem (Merck Schuchardt, Hohenbrunn, Germany) and the N-α-Fmoc-amino acids from Orpegen Pharma (Heidelberg, Germany) and Iris Biotech (Marktredwitz, Germany). The composition of all synthetic substrates (substrates **1**–**13**) is shown in Table 1.

**Table 1:** Synthetic substrate amino acid sequences used in this study [13].

Substrate number	N-term	Substrate sequence	C-term
**1**	K(FITC)	LPETG	amide
**2**	K(FITC)	EGTLP	amide
**3**	K(FITC)	AKKSELPETGGEESTNKGMLFGGLFSILGLALLRRNKKNHK	amide
**4**	K(FITC)	AKKSELPETGGEESTNKRRNKKNHKAGMLFGGLFSILGLALL	amide
**5**	K(FITC)	AKKSELPETGGEESTNKRRNKLKNHK	amide
**6**	K(FITC)	LPETGGEESTNKRKKW	amide
**7**	K(FITC)	LPMTG	amide
**8**	K(FITC)	K(Vancomycin)LPMTG	amide
**9**	K(FITC)	K(Vancomycin)MGTLP	amide
**10**	K(FITC)	LPMTGG-K(vancomycin)	amide
**11**	K(FITC)	Vancomycin-K-AKKSELPETGGEESTNKRRNKLKNHK	amide
**12**		K(Vancomycin)LPMTG	amide
**13**		K(Vancomycin)MGTLP	amide

### FITC- labelling

Peptides were labelled with FITC (Fluorescein isothiocyanate) as the final step of the solid-phase synthesis by coupling FITC to the ε-aminogroup of the N-terminal lysine residue which was introduced using Boc-Lys(ε Fmoc)-OH [14].

### ‘Click’ addition of vancomycin

A previously-described alkyne-modified vancomycin building block was prepared by coupling *N*-(3-(2-(2-(3-aminopropoxy)ethoxy)ethoxy)propyl)pent-4-ynamide to the vancomycin C-terminus [18]. *N*-(3-(2-(2-(3-aminopropoxy) ethoxy)ethoxy)propyl)pent-4-ynamide was prepared by first mono-Boc protecting one amino group of 4,7,10-Trioxa-1,13-tridecanediamine with 1 equiv. Boc according to literature protocols [19] after which 4-pentynoic acid was coupled [20]. Removal of the Boc group was accomplished using standard conditions (1:1 trifluoroacetic acid/ dichloromethane (TFA/DCM), 1 hr) to provide the TFA salt of the desired product as a light yellow oil that was used directly after evaporation of TFA and DCM. Vancomycin (AppliChem, Darmstadt, Germany) was conjugated to the amino-alkyne spacer as follows: *N*-(3-(2-(2-(3-aminopropoxy) ethoxy)ethoxy)propyl)pent-4-ynamide (250 mg, 0.63 mmol, 3 equiv.) was dissolved in DMF (10 ml) followed by addition of triethylamine (351 μl, 2.52 mmol, 4 equiv.), vancomycin-HCl (936 mg, 0.63 mmol, 1.0 equiv.), EDAC (133 mg, 0.69 mmol, 1.1 equiv.) and HOBt (102 mg, 0.76 mmol, 1.2 equiv). The reaction mixture was stirred at ambient temperature for 2 days after which the DMF was removed in a vacuum concentrator (CHRIST, Osterode am Harz, Germany) and the residue was purified using preparative RP-HPLC as described below for peptide purification. The vancomycin-alkyne conjugate was next coupled to the azide-containing peptide by means of Cu(I) catalyzed ‘click’ chemistry following a procedure described previously [21]. The azido peptide was prepared by introducing L-azidolysine (Fmoc-Lys(N_3_)-OH) (obtained from Chiralix, Nijmegen, The Netherlands). To perform the ‘click’ reaction, the vancomycin alkyne was added in equimolar concentrations to the resin-bound azido peptide in a 20 ml syringe, containing DMF (5 ml), 2 equiv. of 2,6-lutidine, 2 equiv. 2,2-bipyridine, 1 equiv. CuBr and 2 equiv. of sodium ascorbate (all peptide grade, Actu-All Chemicals) and flushed for 1 min with N_2_. After incubation for 24 hrs at ambient temperature the mixture was flushed sequentially with DMF, H_2_O, MeOH, EDTA (100 mM), H_2_O, and DMF followed by washing and drying by flushing three times with isopropanol and DCM each. Next the peptide was detached from the resin and purified as described below for peptide purification.

### Peptide purification

Peptides were purified by preparative RP-HPLC (Dionex Ultimate 3000, Thermo Scientific, Breda, The Netherlands) on a Grace Spring column (250 mm x 25 mm, Grace, Deerfield, IL, USA) containing Vydac C18 TP beads,10 μm (Vydac, Hesperia, CA, USA). Elution was performed with a linear gradient from 15 to 45% AcN containing 0.1% TFA in 20 min at a flow rate of 20 ml/min. The absorbance of the column effluent was monitored at 214 nm, and peak fractions were pooled and lyophilized. Re-analysis by RP-HPLC on an analytic Vydac C18-column (218MS54) developed with a similar gradient at a flow rate of 1 ml/min revealed a purity of at least 95%. The authenticity was confirmed by mass spectrometry with a Microflex LRF MALDI-TOF, equipped with a gridless reflectron (Bruker Daltonik GmbH, Bremen, Germany).

### Bacterial strains

The *S*. *aureus* 8325–4 wild type (WT) strain and its isogenic *srtA* deletion mutant (*srtA* KO) were used in this study. The *srtA* mutant was generated by *srtA*:*ermC* allele transduction, as previously described [9, 13]. The *srtA* KO mutant was selected and maintained on brain-heart infusion agar (BHA, BD (Becton Dickinson and Company)-Difco, Etten-Leur, The Netherlands) plates supplemented with 3 μg/ml erythromycin (Abbott Laboratories, North Chicago, Illinois, U.S.).

### *In situ* incorporation of SrtA substrates

WT and *srtA* KO strains were grown overnight on respectively BHA and BHA supplemented with 3 μg/ml erythromycin. Subsequently, bacteria were resuspended in Luria-Bertani medium (LB, BD-Difco, Etten-Leur, The Netherlands), to an OD_600nm_ of 0.1. Twenty-five microliters of bacterial suspension was mixed with 25 μl LB containing up to 1 mM substrate (Table 1). Bacteria were then cultured in 96 wells-plates until the late stationary phase (approximately 24 hrs) in the dark at 37°C with continuous shaking at 230 rpm. As additional controls, we used 0.5 or 1 μM vancomycin and a combination of vancomycin with substrate **7** at 0.5 or 1 μM.

### Flow cytometry

The bacteria were washed four times with PBS followed by centrifugation for 5 min at 3,700 x *g*. To remove the non-covalently bound and intracellular substrate, bacteria were washed with 1% of sodium dodecyl sulphate (SDS, Merck-Schuchardt OHG, Hohenbrunn, Germany) at 60°C for 5 min. Subsequently, bacteria were washed four times with PBS followed by centrifugation for 5 min at 3,700 x *g*. Labelled bacteria were resuspended in 100 μl of 4% formaldehyde (Sigma-Aldrich, Zwijndrecht, The Netherlands) in PBS and incubated for 45 min at ambient temperature with continuous agitation. After 3 washes with PBS, the bacteria were resuspended in 0.5 ml PBS. The bacteria-associated fluorescence was measured on a FACS Canto II^™^ flow cytometer and analysed using FacsDiva^™^ software (BD Biosciences, Breda, The Netherlands) [13].

### Effect of growth phase on substrate 5 incorporation

Substrate **5** (Table 1) incorporation was characterized in greater detail due to its enhanced incorporation levels. Bacteria were cultured in LB medium in the continuous presence of either (i) 1 mM substrate **1** or (ii) 250 μM substrate **5** until either a) exponential growth phase (OD_600nm_ 0.5), b) post-logarithmic growth phase (OD_600nm_ 1), c) late exponential growth phase (OD_600nm_ 1.2) or d) late stationary growth phase (24 hrs incubation) in the dark at 37°C with continuous shaking (230 rpm). After the various growth phases had been reached, bacteria were harvested and the incorporation of substrates was established by determining the presence of FITC using FACS analysis.

In another experiment, bacteria were cultured (LB medium) in the absence of substrate to the late stationary growth phase. Subsequently the bacteria were diluted to an OD_600nm_ of 0.5, washed 3 times with PBS, and then incubated with either; i) SrtA buffer (50 mM Tris, 150 mM NaCl, 10 mM CaCl_2_, pH 7.5), ii) substrate **1**, or iii) substrate **5** (Table 1) in SrtA buffer. Bacteria were incubated in the presence of 0 to 1 mM of these substrates using a final volume of 50 μl and 96 wells-plates. Incubation was performed for 17 hrs in the dark at 37°C with continuous shaking (230 rpm).

### Determination of the minimal inhibitory concentration (MIC) of vancomycin and vancomycin-conjugates

WT and *srtA* KO bacteria were cultured in LB medium in a 96-wells plate in the presence of serial dilutions (0.1 to 100 μM) of either vancomycin (Sigma-Aldrich, Zwijndrecht, The Netherlands) or substrate **8**; **9**; **12** or **13** at 37°C with continuous shaking (230 rpm). After 24 hrs, the wells were visually inspected and the first clear well was designated as the MIC.

All experiments were repeated independently three times and the standard error of the mean (SEM) was calculated.

### Statistical analysis

Statistical analysis was performed using the Prism 5.0 package (GraphPad software, San Diego, CA, USA). We used one-way ANOVA testing with Bonferroni correction, considering P < 0.05 as being statistically significant.

## Results

### Optimization of synthetic SrtA substrates

**Fig 1:**
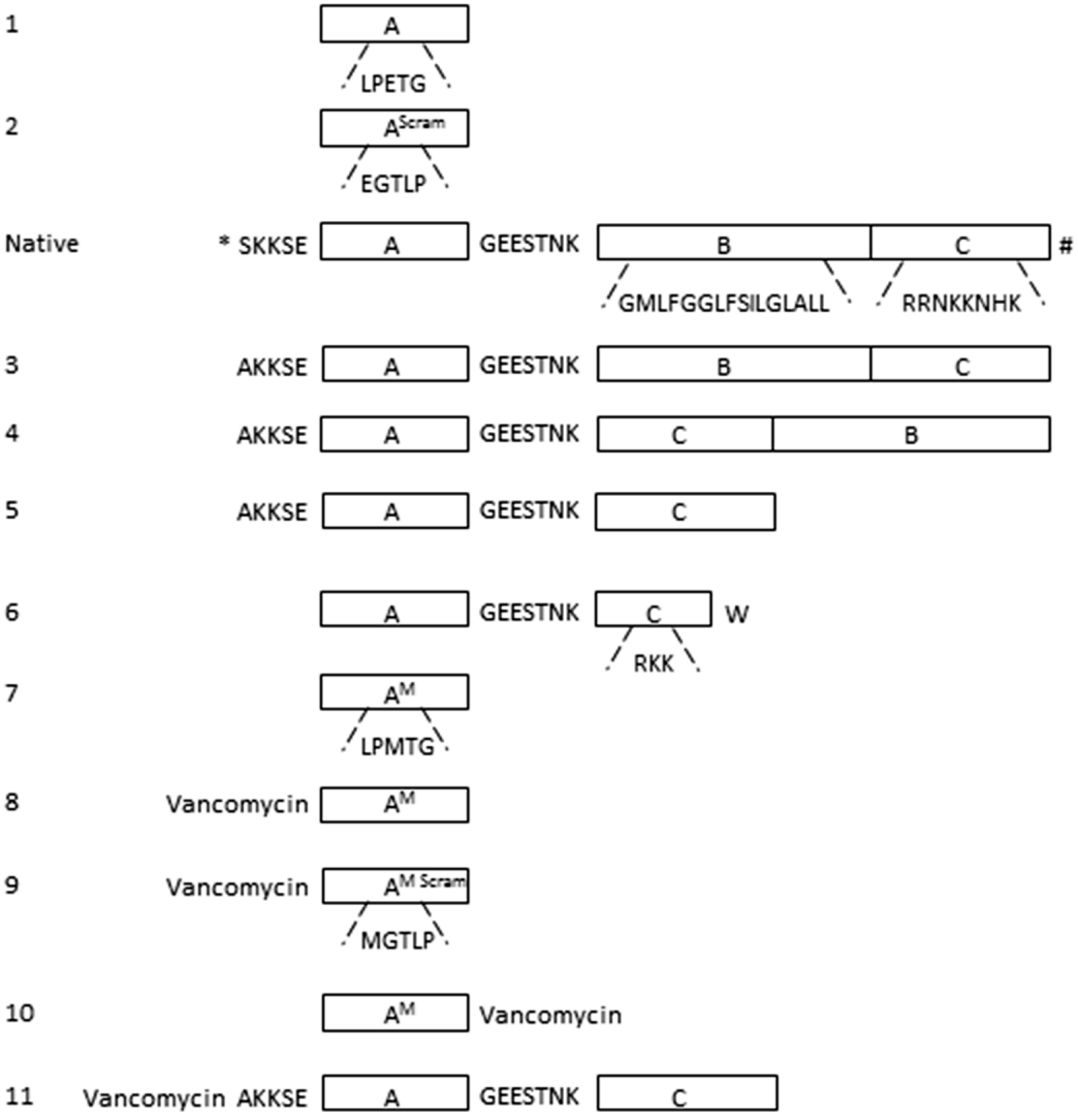
Schematic representation of the C-terminal region of FnBP precursor and synthetic substrates used in this study. **1**: the minimal substrate used, containing the SrtA recognition motif LPETG only (designated A). **2**: the scrambled version of substrate **1**, EGTLP. **Native**: the sorting motif of the FnBP precursor of *S*. *aureus*, containing the SrtA recognition motif (A), a hydrophobic domain (B) and a positively charged region (C). The full sequences of the separate regions are depicted under the blocks. Domain (A) and (B) are linked via a hydrophilic spacer GEESTNK [24]. The N-terminus and the C-terminus are indicated with * and # respectively. **3**: schematic representation of substrate **3**, based on the complete sorting signal of the FnBP precursor. **4**: schematic representation of substrate **4**, in which domain (B) and (C) are interchanged. **5**: schematic representation of substrate **5**, in which domain B is removed. **6**: schematic representation of substrate **6** containing domain (A), truncated domain (C) and a C-terminal tryptophan. **7**: schematic representation of substrate **7** containing domain (A), with glutamate (E) substituted for methionine (M). **8**: schematic representation of substrate **8**, composed of substrate **7** with its N-terminus linked to vancomycin. **9**: schematic representation of substrate **9**, the scrambled version of substrate **8**, with an MGTLP motif instead of an LPMTG motif. **10**: schematic representation of substrate **10**, composed of substrate **7** with its C-terminus linked to vancomycin. **11**: schematic representation of substrate **11**, composed of substrate **5** with its N-terminus linked to vancomycin. All substrates were at the N-terminus labelled with a FITC group.

**Fig 2:**
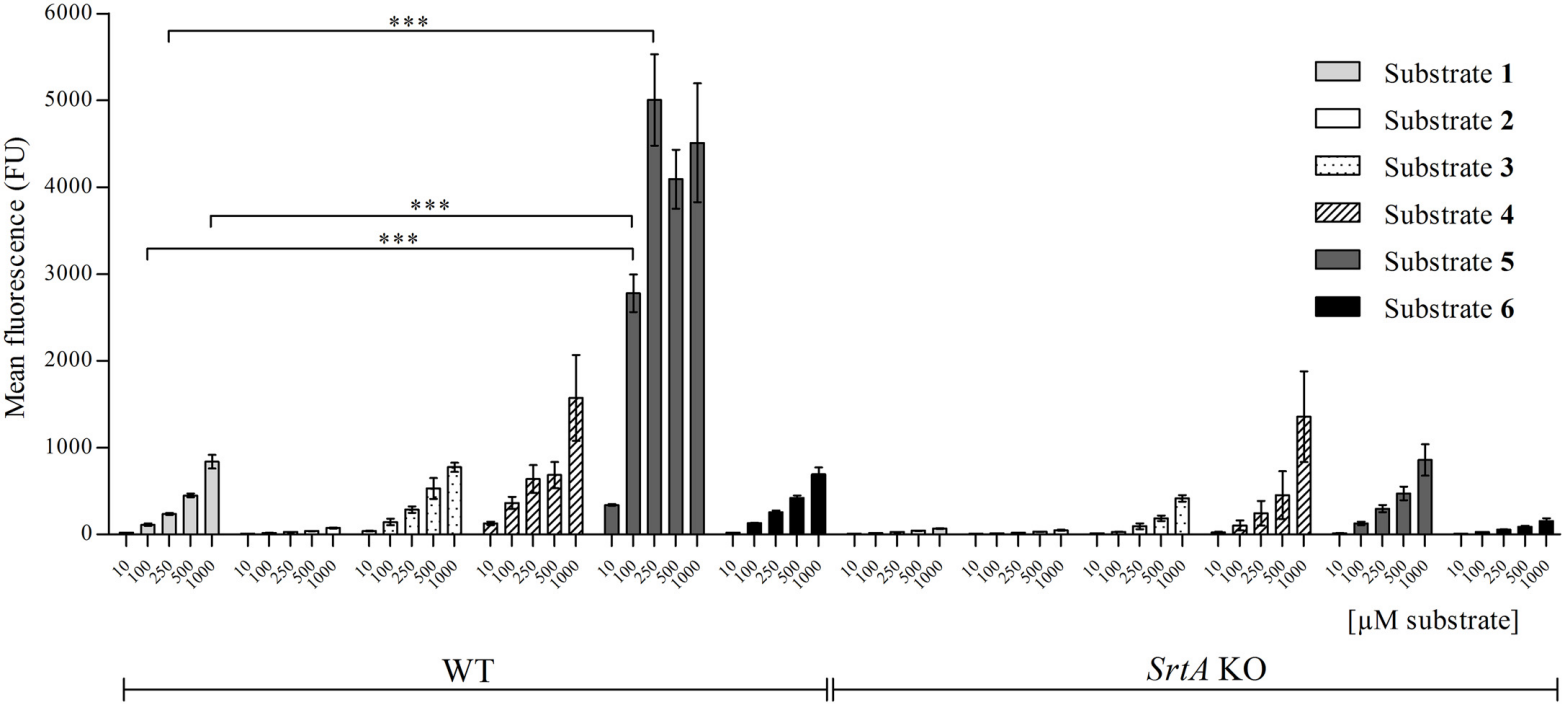
Titrations of the SrtA synthetic substrates. Cultures of 8325–4 WT and *srtA* KO strains were incubated in LB medium during 24 hrs in the presence of either substrate **1**; **2**; **3**; **4**; **5** or **6** [13] at 10 μM, 100 μM, 250 μM, 500 μM or 1 mM and analysed by FACS. The synthetic substrate concentrations used are depicted on the X-axis and the mean fluorescence, as a measure for the level of substrate incorporation into the bacterial cell wall, was plotted on the Y-axis. Substrate **1** signal at 100 μM and 250 μM was significantly lower than the signals obtained with substrate **5** at the same concentration (P < 0.0001). Substrate **1** signal obtained at 1 mM was significantly lower than the signal obtained with substrate **5** at 100 μM (P < 0.0001). Only the most relevant significant differences are presented.

*In vivo* the incorporation of physiological SrtA substrates is determined by a recognition signal in their C-terminus. The C-terminus contains three motifs: (A) the LPXTG SrtA recognition signal, (B) a hydrophobic transmembrane domain and (C) a cluster of cationic amino acid residues (See Fig 1, native substrate). The LPXTG motif protrudes from the outside of the bacterium, and is linked via a hydrophilic negatively charged spacer GEESTNK to the transmembrane domain, which in turn is connected to the cationic cluster at the cytosolic side of the membrane [4, 22, 23]. In a previous study, we found that an artificial motif (K(FITC)LPETG) (substrate **1**, Fig 1, Table 1) is covalently incorporated in a SrtA-dependent manner into the cell wall of *S*. *aureus* [13]. In the present study, we first tested a series of artificial SrtA substrates equipped with the additional domains B and C, to examine the effect on incorporation. To monitor their incorporation in the cell wall by FACS, the substrates were linked via an additional lysine (K) residue to FITC. *S*. *aureus* WT and its isogenic *srtA* KO (control) were cultured in the presence of substrates for 24 hrs. After washing, to remove non-covalently bound substrates, the cell-associated fluorescence was determined by FACS analysis. A scrambled version of substrate **1** (K(FITC)EGTLP, substrate **2**, Fig 1, Table 1) was used as a negative control. *S*. *aureus* (WT) cultured in the presence of 1 mM substrate **1** showed a mean fluorescence level of 840 FU (Fluorescence Units). In the *srtA* KO strain little if any incorporation of substrate **1** was observed (70 FU). Culturing in the presence of the scrambled substrate **2** did not result in any substantial incorporation, neither in the WT nor in the *srtA* KO strain (75 FU and 50 FU for WT and *srtA* KO strains, respectively) (Fig 2). This confirms previous studies [13, 14] that the incorporation of substrate **1** was mediated for > 90% by SrtA. We next tested a set of substrates that included the native sequence of the C-terminal region of the fibronectin-binding protein (FnBP) of *S*. *aureus* (substrate **3**, Fig 1, Table 1) [24]. In substrate **3** (AKKSELPETGGEESTNKGMLFGGLFSILGLALLRRNKKNHK), the LPETG motif is separated from the hydrophobic (GMLFGGLFSILGLALL) and cationic (RRNKKNHK) domains by the negatively charged sequence GEESTNK, which functions as a hydrophilic spacer. Substrate **3** is flanked at the N-terminal part with a sequence of non-repetitive region of cell wall-spanning domain AKKSE to increase the targeting towards the cell-wall. Incorporation of substrate **3** in *S*. *aureus* WT was comparable to that observed with substrate **1**, with a mean fluorescence intensity per cell of 800 FU at 1 mM. In contrast to substrate **1**, substrate **3** labelled also the *srtA* KO strain (400 FU at 1 mM) (Fig 2).

The position of the motifs A, B and C in substrate **3** is tailored for transferring physiological SrtA substrates from the inside to the outside of the bacteria *in situ*. This configuration may therefore be sub-optimal for the incorporation of exogenous substrates, which target the endogenous SrtA from the *outside*. We therefore examined whether interchanging the positions of the membrane-spanning domain (B) and the cationic cluster (C) (substrate **4**, Fig 1, Table 1) would result in a higher incorporation. This peptide, however, labelled *S*. *aureus* WT and *S*. *aureus srtA* KO to the same extent (1600 versus 1400 FU) (Fig 2). Substrates **3** and **4** formed precipitates during incubation, due to their hydrophobic character, which could not be completely removed in the washing steps and stayed attached to the bacteria. We envisage that this has caused the SrtA-independent background labelling of the *S*. *aureus srtA* KO strain. Because of the poor solubility, substrates **3** and **4** were not further examined. We next tested substrate **5** (Fig 1, Table 1), which lacks the hydrophobic membrane-spanning domain (B). This substrate was incorporated at a higher efficiency compared to substrate **1** (Fig 2). Between 10 and 250 μM the incorporation of substrate **5** increased almost linearly with the concentration. In this concentration range, substrate **5** showed an approximately 20-fold higher incorporation than substrate **1**. At concentrations > 250 μM, the incorporation of substrate **5** levelled off, suggesting that the process became saturated. Substrate **5** revealed a higher background in *srtA* KO strain than substrate **1** (860 vs 70 fluorescence units at 1 mM, Fig 2). This is probably caused by SrtA independent electrostatic interactions between the cationic cluster and the bacterial cell surface. Still, the incorporation of substrate **5** in the *S*. *aureus* WT strain was 10 to 20-fold higher than in the *S*. *aureus srtA* KO strain, indicating that > 90% of the incorporation of substrate **5** was mediated by SrtA, similar to that of substrate **1**. In an attempt to further minimize the substrate, a variant (LPETGGEESTNKRKKW (substrate **6**, Fig 1, Table 1)) was developed composed of the SrtA recognition motif LPETG, linked via the GEESTNK linker to a minimal cationic domain C (RKK) and terminated at the C-terminus by a tryptophan (W) residue. As this residue has a preference for the membrane-water interface [25, 26], we reasoned that it might target the substrate to the bacterial membrane. The efficiency of this peptide, however, did not differ from that of substrate **1** (700 FU; 1 mM), with the background labelling of 160 FU at 1 mM (Fig 2).

### Effect of growth phase on substrate incorporation

**Fig 3:**
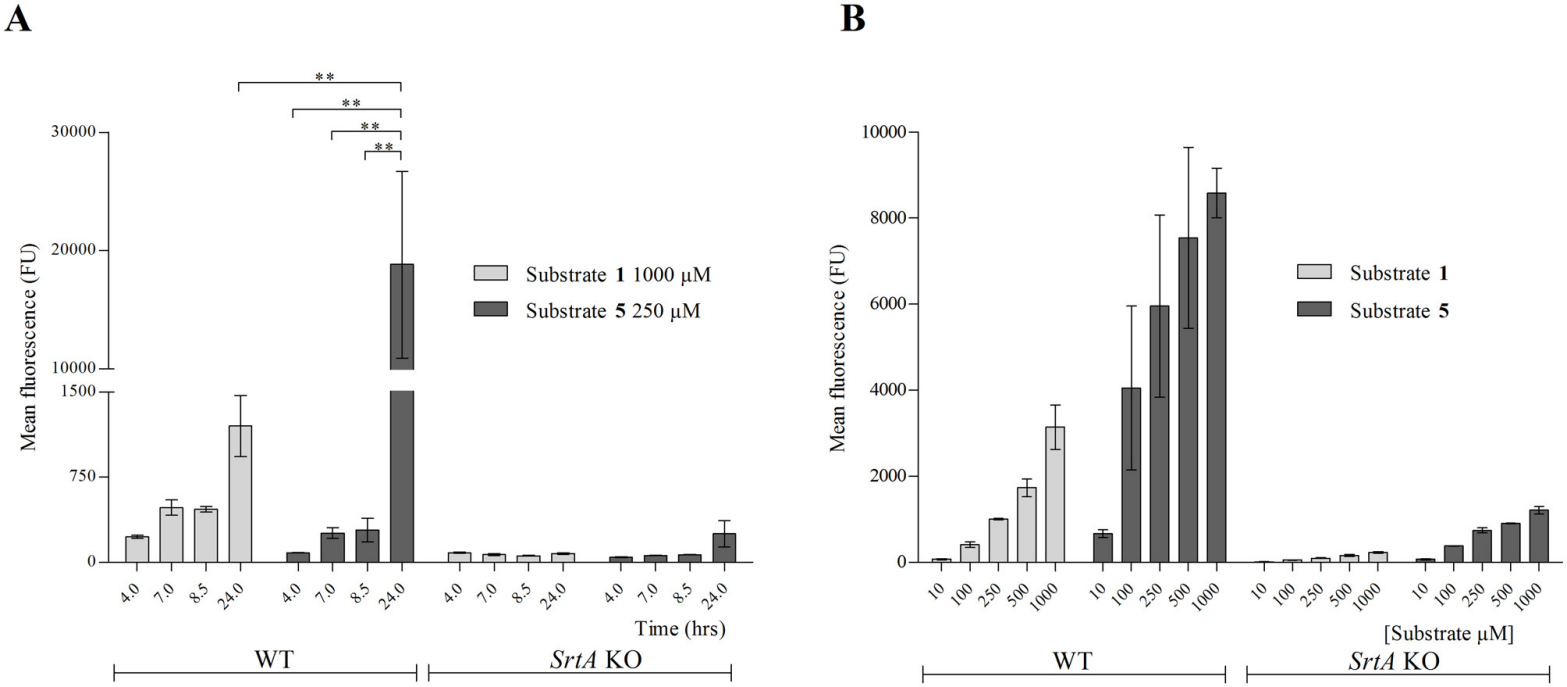
Kinetics determination of substrate 5. 8325–4 WT and *srtA* KO strains were incubated in the presence of (A) either 1 mM of substrate **1** or 250 μM of substrate **5** until either exponential (4 hrs), post-logarithmic (7 hrs), late exponential (8.5 hrs) or late stationary phase (24 hrs) in LB medium. The mean fluorescence of substrate **1** and substrate **5** is depicted on y-axis. (B) Washed and diluted (OD_600nm_ 0.5) late stationary grown WT and *srtA* KO bacteria were incubated with either 10 μM, 100 μM, 250 μM, 500 μM or 1 mM substrate **1** or substrate **5** in SrtA buffer during 17 hrs. The mean fluorescence was determined by FACS. The signal obtained with substrate **1** at 1 mM after 24 hrs of incubation was significantly lower than the signal obtained with substrate **5** at 250 μM after 24 hrs (P < 0.001). Substrate **5** signals at time points 4 hrs, 7 hrs and 8 hrs were all significantly lower in comparison to the signal obtained with the same substrate at 24 hrs time point at 250 μM (P < 0.001).

To analyse the effect of growth phase on the cumulative incorporation of the SrtA substrates, *S*. *aureus* (WT and *srtA* KO strains) were cultured in the presence of substrate **1** and **5**, at a concentration that yielded the maximal incorporation level (1 mM and 250 μM, respectively). Bacteria were harvested after 4 hr (exponential phase), 7 hr (post-logarithmic phase), 8.5 hr (late exponential phase) and 24 hr (late stationary phase) and analysed by FACS (Fig 3A). After 8.5 hrs both substrates showed comparable incorporation (approximately 500 and 300 FU, respectively). Between 8.5 and 24 hrs the incorporation of substrate **1** increased approximately three-fold, whereas that of substrate **5** increased more than 60-fold (from 300 to 19,000 FU; Fig 3A). We then explored the incorporation of substrate **5** in late stationary *S*. *aureus*. To do so, *S*. *aureus* was cultured until late stationary phase in the absence of substrates. After washing, bacteria were transferred into SrtA reaction buffer (conditions where the bacterial metabolism is suppressed) supplemented with either substrates **1** or **5** at concentrations varying between 10 μM to 1 mM. After 17 hrs, the reaction was stopped and incorporation was determined by FACS; bacteria treated with substrate **5** displayed a 4 to 10 times higher fluorescence than those incubated with substrate **1** (Fig 3B). This supported that substrate **5** was incorporated with a higher efficiency than substrate **1**.

### Effect of glutamate to methionine substitution

**Fig 4:**
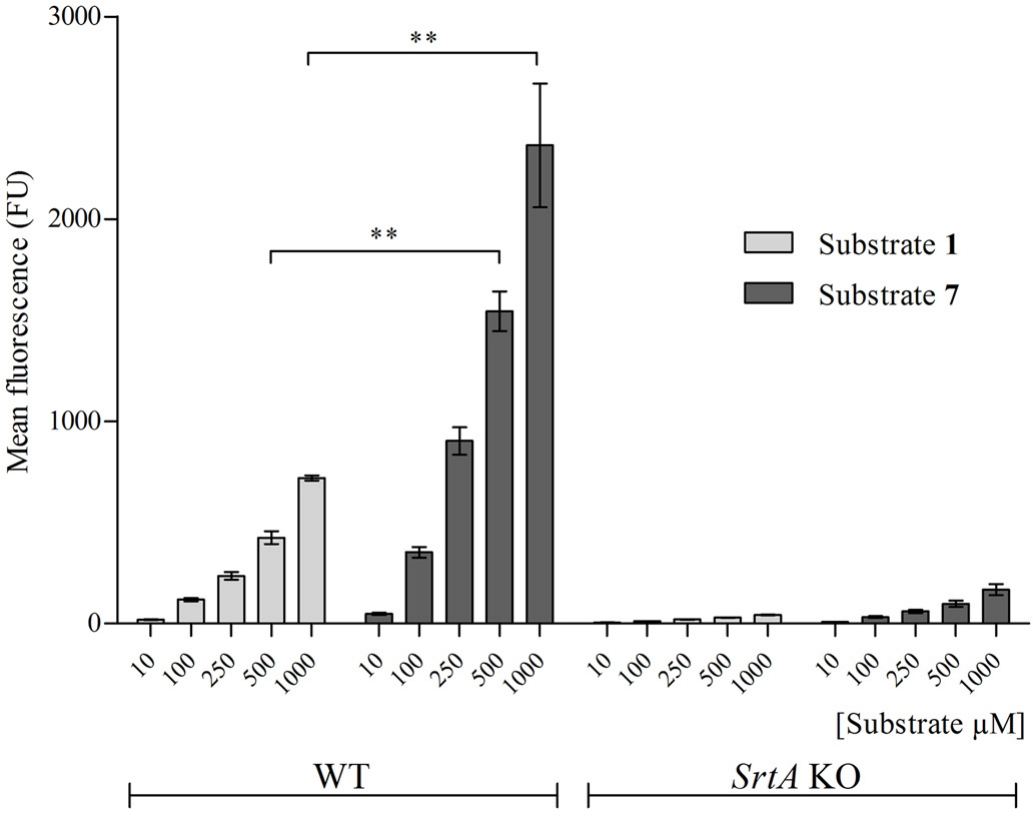
Incorporation of substrate 7. WT and *srtA* KO *S*. *aureus* bacteria were incubated in the presence of either substrate **1** or substrate **7** at 10 μM, 100 μM, 250 μM, 500 μM or 1 mM concentrations in LB medium during 24 hrs and analysed by FACS. The incorporation of substrate **7** was significantly higher at 500 μM and at 1 mM compared to substrate **1** at the same concentrations after 24 hrs in WT bacteria (P < 0.001).

The third position in the SrtA recognition motif (LP*X*TG) is tolerant to various amino acids substitutions. Still, some residues are preferred over others, *e*.*g*. LPMTG is more rapidly processed than LPETG by isolated recombinant SrtA *in vitro* [15]. Therefore, we examined if substituting E for M (substrate **7**, Fig 1, Table 1) would also enhance the *in situ* processing of exogenous SrtA substrates. Substrate **7** was incorporated significantly better than substrate **1** into the WT bacterial surface at 500 μM and 1 mM (Fig 4). Substrate **7** showed little if any incorporation in *srtA* KO strain (150 FU), indicating that its incorporation in *S*. *aureus* WT required a functional SrtA.

### Effect of vancomycin conjugation on incorporation

**Fig 5:**
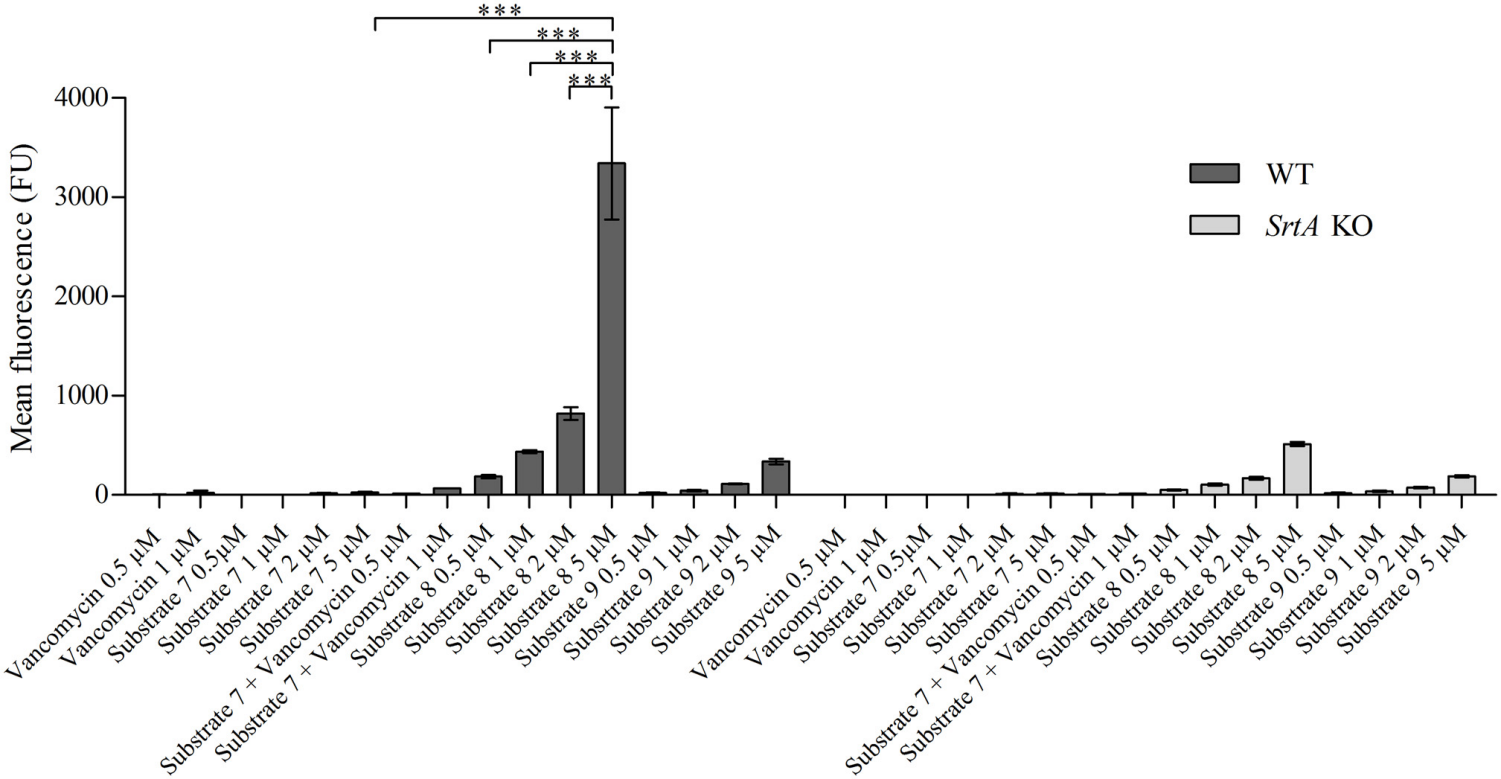
Incorporation of vancomycin—conjugated substrate. 8325–4 WT and *srtA* KO bacteria were incubated in the presence of either 0.5 μM or 1 μM of 1) vancomycin; 2) substrate **7** + vancomycin; or 0.5 μM; 1 μM; 2 μM or 5 μM of 3) substrate **7**; 4) substrate **8** or 3) substrate **9** in LB medium during 24 hrs and analysed by FACS (mean fluorescence). (***: P < 0.0001).

The glycopeptide antibiotic vancomycin targets the D-ala-D-ala motif of lipid II, which *in vivo* functions as an acceptor for SrtA-processed CWA proteins. Vancomycin binds specifically to lipid II and decreases its accessibility, thereby interfering with SrtA-dependent anchoring of CWA proteins [7, 27, 28]. Conjugation of vancomycin to substrate **7** was therefore considered as an attractive strategy to enhance the targeting of these substrates to the vicinity of SrtA. To do so, vancomycin was coupled to substrate **7** at either the penultimate N-terminal position to yield K(FITC)K(vancomycin)LPMTG (substrate **8**, Fig 1, Table 1) or the C-terminal site of the peptide, to yield K(FITC)LPMTGGK(vancomycin) (substrate **10**, Fig 1, Table 1). As a control served substrate **9** (K(FITC)K(vancomycin)MGTLP), a scrambled variant of substrate **8** (Fig 1, Table 1). The *in situ* incorporation of the vancomycin-LPMTG conjugates was assessed by culturing *S*. *aureus* in LB medium supplemented with the substrates at concentrations varying from 0.5 μM—5 μM (Fig 5). This revealed that the vancomycin-modified substrates **8** and **10** (the latter not shown) were incorporated to the same extent as the parent peptide **7**, but at a 200-fold lower concentration (Fig 5). Culturing in the presence of equimolar concentrations of vancomycin and substrate **7** did not result in labelling of *S*. *aureus*, ruling out that vancomycin by itself stimulated the incorporation of substrate **8** and **10** (Fig 5). At the highest concentration tested (5 μM), background labelling of substrate **8** with the *srtA* KO strain was observed, possibly due to formation of non-covalent complexes of vancomycin with its D-ala-D-ala ligand. Similarly, the control substrate **9** also showed background staining with both the WT and the *srtA* KO strain at higher substrate concentration. The specific substrate incorporation of substrate **8** in WT bacteria was 10- fold higher than in the *S*. *aureus srtA* KO strain incubated with substrate **8** and *srtA* KO and WT strains incubated with control substrate **9**. These data indicate that > 90% of the incorporation of substrate **8** was mediated by SrtA. Conjugation of vancomycin to substrate **5** (substrate **11**, Fig 1, Table 1) did not result in the same increased incorporation observed for conjugates **8** and **10** (data not shown).

**Fig 6:**
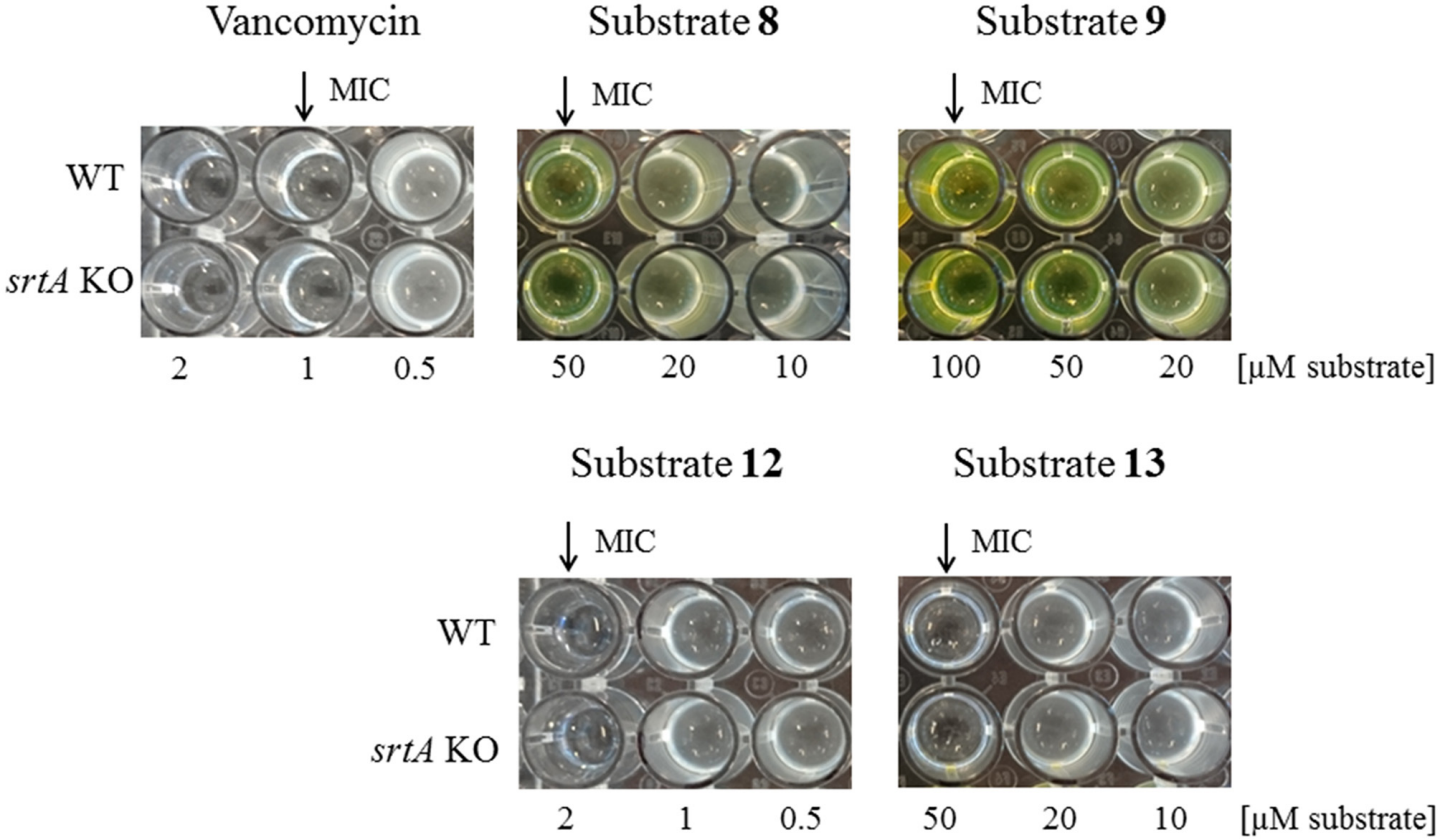
MIC determination of vancomycin-conjugated substrates. WT and *srtA* KO bacteria were cultured in LB medium in the presence of serial dilutions (0.1 to 100 μM) of either vancomycin or substrate **8**; **9**; **12** or **13** at 37°C with continuous shaking (230 rpm) during 24 hrs. The MIC-value (first clear well) was visually determined after 24 hrs and is indicated with an arrow.

Apparently, coupling of LPMTG to vancomycin enhanced the processing by SrtA. We wondered if, reciprocally, LPMTG would enhance the targeting of vancomycin to lipid II, and henceforth its antibacterial activity. Determination of the MIC-values of substrate **8** and **9**, however, showed that conjugation of vancomycin with LPMTG negatively affects its antimicrobial activity, causing an increase in the MIC of vancomycin from 1 μM to either 50 or 100 μM for substrate **8** and **9**, respectively, in both the WT and the *srtA* KO strain (Fig 6). To test the effect of vancomycin modification on MIC increase within the substrate **8** and **9**, we generated substrate **12** and **13** lacking the FITC molecule. The MIC of these non-FITCylated variants were 2 μM (substrate **12**) and 50 μM (substrate **13**) (Fig 6). These data suggest, that the addition of FITC molecule decreases the antimicrobial activity of the vancomycin unit within the synthetic substrate.

## Discussion

In the present study, we have examined the effect of various modifications on the incorporation efficiency of artificial SrtA substrates. Physiological SrtA substrates contain, besides the LPXTG motif, a hydrophobic transmembrane domain and a cluster of positively charged amino acids, which together retain the protein in the vicinity of the bacterial membrane [4]. *In vivo*, this prevents leakage of key CWA surface proteins into the bacterial surroundings, and facilitates efficient processing by SrtA. By elongating the LPXTG motif with these domains, we aimed to improve the *in situ* processing of synthetic SrtA substrates. However, substrates **3** and **4** equipped with both the hydrophobic membrane-spanning and the cationic domain, were poorly soluble and formed visible aggregates. This probably caused high non-specific adsorption to bacterial surfaces (see Fig 2). Omitting the hydrophobic domain (substrate **5**), however, resulted in 5-fold better incorporation than the parent substrate. Also substrate **5** exhibited more a-specific binding to the *srtA* KO strain than the parent peptide **1**, probably due to a strong electrostatic binding with the bacterial surface. The incorporation of substrate **5** reached saturation at concentrations ≥ 250 μM. This suggests that under these conditions another factor, likely the number of accessible pentaglycine acceptors, had become limiting [29, 30]. Strikingly, even in the presence of these saturating concentrations of exogenous substrate, the cell-wall expression of the endogenous substrate SpA was not decreased (Hansenova Manaskova *et al*, unpublished observations), indicating that this physiological substrate is preferentially processed by SrtA. In line, incorporation of SpA predominantly occurs in the exponential growth phase [31], whereas incorporation of substrates **5** (Fig 3) and substrate **1** [13] mainly takes place in the stationary phase, when the synthesis of CWAs is subdued [31].

Substitution of E by M within the LPXTG motif (substrate **7**) resulted in a 3-fold higher incorporation. This is in a close agreement with the results of a previous study, which found that LPMTG *in vitro* is cleaved by recombinant SrtA approximately 2.5 times faster than LPETG [15]. Interestingly, the SrtA recognition motifs of different native CWA proteins contain various different amino acids at the ambiguous third position: lysine, glutamic acid, aspartic acid, alanine or glutamine, but none of them contains methionine [32].

Conjugation of LPMTG to vancomycin enhanced the incorporation efficiency drastically: the same level of incorporation was obtained at 200- to 500-fold lower concentrations than with the unconjugated substrate (Fig 5). SrtA-independent labelling of the *S*. *aureus* WT and the *S*. *aureus srtA* KO strain was observed with substrate **8** and the scrambled substrate **9**, possibly caused by vancomycin direct binding to D-ala-D-ala. Interestingly, conjugation of the peptide moiety to vancomycin increased the MIC value 50- to 100-fold, in line with similar decreases in antibiotic activity observed for other fluorescently-labelled vancomycin probes, which despite a >100-fold increase in MIC value, labelled the bacterial cell wall at sub-MIC concentrations [33]. The fluorescent label in vancomycin substrates seems to be responsible for the MIC increase, as substrate **12** (substrate **8** variant without FITC) had comparable MIC value to vancomycin molecule only (2 μM and 1 μM, respectively).

In summary, we have demonstrated that modification of SrtA synthetic substrates is a feasible approach to enhance their *in situ* incorporation by endogenous *S*. *aureus* SrtA. Interestingly, we found that vancomycin-LPMTG conjugates are incorporated in the cell wall of *S*. *aureus* with high efficiency. Such molecules may form a platform for targeted modification of the bacterial surface for e.g. antimicrobial or imaging purposes.
